# Clinical and genotypic analysis of 79 children with methylmalonic acidemia: a retrospective single-center study in China

**DOI:** 10.3389/fendo.2026.1806231

**Published:** 2026-06-15

**Authors:** Huimin Yu, Xiangbo Xie, Chen Chen, Donglei Chen, Yan Sun, Guimei Li, Xiaohong Shang, Jianmei Yang

**Affiliations:** 1 Department of Pediatric Endocrinology, Shandong Provincial Hospital Affiliated to Shandong First Medical University, Jinan, Shandong, China; 2 Shandong University of Traditional Chinese Medicine, Jinan, Shandong, China; 3 Endocrinology and Metabolism, School of Biomedical Sciences (SBMS), University of Queensland, St Lucia, QLD, Australia

**Keywords:** clinical manifestations, gene mutation, homocysteine, methylmalonic acidemia (MMA), prognosis

## Abstract

**Objective:**

Methylmalonic acidemia (MMA) is a common hereditary disorder affecting infants and children. This study investigated the onset age, phenotypes, genotypes, and biochemical features of MMA in 79 children from a single center in Shandong Province, providing data to aid early diagnosis and management.

**Methods:**

The data were obtained from 79 children who were diagnosed with MMA through genetic analysis from January 2014 to December 2023. A retrospective analysis of the age of onset, clinical manifestations, biochemical characteristics, imaging features, gene mutations, and hospitalization status was performed.

**Results:**

Among the 79 patients, 47 had combined MMA with hyperhomocysteinemia, whereas 32 had isolated MMA. Symptoms occurred at any age, with some patients exhibiting symptoms within days after birth. Early-onset patients exhibited more severe symptoms and worse prognosis. The patients exhibited symptoms of delayed growth and development, motor disorders, intellectual disability, vomiting, and feeding difficulties. Most of the patients were prone to infection. A greater frequency of vomiting was reported among the isolated MMA patients. Echocardiography demonstrated that patients with comorbidities were more likely to develop cardiac abnormalities. MRI revealed more frequent abnormal white matter in the central nervous system in patients with combined MMA with hyperhomocysteinemia. Isolated MMA patients often demonstrated multiple brain abnormalities and severe symptoms. Blood biochemical analysis revealed that MMA patients exhibited various abnormal blood biochemical indicators, among which isolated MMA patients more often demonstrated hyperglycemia. Moreover, 89 mutation sites were detected in the combined MMA with hyperhomocysteinemia, with a total of 23 types being identified. Additionally, the *c.609G>A* mutation was the most prevalent. The isolated MMA demonstrated 64 gene mutation sites, with a total of 29 types being observed. Moreover, the *mut c.729_730insTT* was the most frequently detected mutation. With regular treatment, early diagnosed MMA patients demonstrated significant improvements in their motor and intellectual disabilities.

**Conclusion:**

The symptoms in patients with MMA exhibited significant heterogeneity, with differences among subtypes in clinical characteristics, biochemical abnormalities, and imaging findings. The findings of this study contribute to a better understanding of the clinical features of MMA and provide valuable insights that may aid in improving patient outcomes.

## Introduction

Methylmalonic acidaemia (MMA) is an autosomal recessive organic aciduria that was first recognized as a congenital metabolic disorder in 1967 (1). Its biochemical characteristics arise due to an enzyme deficiency in the conversion of methylmalonyl-CoA to succinyl-CoA (2). Caused by various single-gene mutations, MMA currently lacks curative treatments (3). It represents the most common organic acidemia in China, with Shandong Province exhibiting one of the highest reported incidences nationwide (2). To optimize treatment outcomes for affected children and reduce mortality and disability rates, comprehensive intervention strategies are required. Methylmalonyl-CoA mutase (MMUT, also known as MCM) deficiency is the primary cause of methylmalonic acidemia (MMA), or it may result from vitamin B12 metabolism defects (4). The mitochondrial enzyme MMUT requires 5′-deoxyadenosine cobamide to catalyze the breakdown of branched-chain amino acids, odd-chain fatty acids, and cholesterol, supplying nutrients for the tricarboxylic acid cycle and energy production (5, 6). When MMUT activity is impaired, methylmalonic acid and related toxic metabolites accumulate in tissues and body fluids (7), disrupting the tricarboxylic acid cycle, mitochondrial respiration, and ammonia metabolism (8). This abnormal acid accumulation may damage multiple organs (including the nervous, liver, kidney, and hematopoietic systems) through diverse pathogenic mechanisms (9). Clinical presentation typically occurs shortly after birth, characterized by acute metabolic acidosis, hyperammonemia, vomiting, lethargy, and seizures, which often culminate in severe neurological disabilities (10, 11). Neurological impairment may involve ocular manifestations, presenting as clinical symptoms such as visual inattention and nystagmus (12). Common long-term sequelae include movement disorders, intellectual disability, growth retardation, and neurological impairment (13). The clinical course of MMA is characterised by life-threatening acute metabolic decompensation (AMD). AMD is triggered by catabolic stressors, which induce protein catabolism and increase the burden of toxic metabolites, thereby leading to the onset of clinical symptoms (14). Against this backdrop, although liver transplantation as a therapeutic approach can significantly improve patients’ clinical stability (15), it is subject to various complications including organ availability, surgical risks, and the potential risk of post-operative MMA-related complications (16). Based on the underlying enzyme deficiency, methylmalonic acidemia can be classified into isolated methylmalonic acidemia and compound methylmalonic acidemia with hyperhomocysteinemia. Isolated MMA encompasses the mut_0_, mut^-^, cblA, cblB, and cblH subtypes, among which the mut_0_ subtype represents the most clinically severe phenotype (17–19), whereas compound methylmalonic acidemia, more prevalent in China, frequently involves cblC deficiency (20).

In this study, we analyzed age at onset, clinical manifestations, routine hematologic findings, ancillary investigations, genetic results, and hospitalization patterns during the first five years after diagnosis in children with combined and isolated MMA in Shandong Province. Although several studies have reported on the clinical characteristics of MMA in Chinese children, data from Shandong Province, a region with a significantly higher disease burden, remain limited. The aims of this study were to delineate the disease characteristics in this region, to promote earlier recognition and treatment, and to ultimately reduce long-term morbidity in affected children.

## Subjects and methods

### Subjects

Seventy-nine children with genetically confirmed MMA who visited Shandong Provincial Hospital between January 2014 and December 2023 were enrolled. Based on the presence of hyperhomocysteinaemia and the genetic findings, they were classified into combined MMA with hyperhomocysteinemia (n=47) or isolated MMA (n=32) groups. The cohort included 37 males and 42 females. This was a retrospective study, and all the investigations were performed with written informed consent from the guardians of the children (Ethics approval No. SWYX: No. 2026-216).

### Data collection

Medical records and follow-up information of 79 children treated at Shandong Provincial Hospital between January 2014, and December 2023 were collected. The electronic medical records of all patients were reviewed. All the data were collected from the hospital information system. The clinical information of the patients with MMA consisted of age, sex, course of MMA, body mass index (BMI), family history, clinical manifestations at presentation, ancillary investigations, genetic findings, newborn screening (NBS) status, hospital admission during the first five years after diagnosis, and long-term prognosis. In this study, growth retardation was defined as a height or weight below the 3rd percentile (P3) or 2 standard deviations below the mean (–2SD) of the normal growth standards for children of the same age and sex, as specified in the Growth Standards for Children Under 7 Years of Age (WS/T 423-2022).

### Screening indicators

Total homocysteine (tHCY) concentration was measured via fluorescence polarization immunoassay, and the reference concentration was 0-15 μmol/L. In addition, alanine aminotransferase (ALT), aspartate aminotransferase (AST), serum creatinine, blood urea nitrogen (BUN), blood ammonia, plasma amino acid, and blood lactate levels were measured and collected. Laboratory reference ranges are as follows: Anemia: Hemoglobin in newborns >145 g/L; Children under 6 years old >110 g/L; Children over 6 years old >120 g/L. ALT and AST: 0-40 U/L. Serum creatinine: 24.9-69.7 μmol/L. Blood urea nitrogen: 2.8-7.2 mmol/L. Blood glucose: 3.9-6.1 mmol/L. Hyperlactatemia: Blood lactate >2 mmol/L. Hyperammonemia: Blood ammonia >100 μmol/L.

### Treatment protocol

In this study, a uniform treatment protocol was applied to patients with both isolated and combined forms of MMA. All patients began standardized treatment immediately after a definitive diagnosis, which included: restricting natural protein intake (0.8-1.5 g/kg·d), supplementing with a specialized MMA formula powder free of valine, isoleucine, methionine, and threonine, oral L-carnitine (50-100 mg/kg·d), and vitamin B12 (methylcobalamin or hydroxocobalamin) therapy based on the subtype. For patients with combined-type MMA, betaine was also administered to lower homocysteine levels. During acute metabolic crises, comprehensive treatment should be provided based on the patient’s condition, including intravenous glucose infusion, and correction of acidosis, among others.

### Clinical assessment and outcome measures

In this study, the assessment of clinical outcomes and long-term prognosis were primarily based on the level of symptom control, degree of functional independence, and multisystem involvement. The definition of poor prognosis or disability encompassed severe involvement of multiple systems, with neurological sequelae serving as the primary assessment dimension. These included recurrent metabolic crises accompanied by impaired consciousness, motor dysfunction, and refractory epilepsy. Intellectual disability or developmental regression was also a core criterion for assessment, evaluated based on Wechsler Intelligence Scale scores, the child’s ability to perform activities of daily life and communication, and the severity of learning difficulties. From a neuroimaging perspective, structural brain injuries such as basal ganglia lesions, progressive brain atrophy, and white matter lesions identified via MRI were all regarded as objective indicators of an unfavorable prognosis. Furthermore, dysfunction in organs beyond the central nervous system served as another critical criterion for poor outcomes, with particular emphasis on chronic kidney disease resulting from progressive renal failure, cardiomyopathy, and recurrent pancreatitis. In terms of growth and metabolic assessment, another indicator of poor prognosis was defined as two or more hospitalizations per year due to acute metabolic decompensation, accompanied by severe growth retardation.

In contrast, clinical improvement was defined as the achievement of specific functional milestones and symptom stabilization, rather than a complete return to age-appropriate developmental levels. Specific evaluation criteria included: effective control of clinical symptoms, manifested by reduced muscle tone, decreased tremors or abnormal movements, and reduced or complete control of seizure frequency over a significant period. Functional benefits were reflected in improvements in the child’s motor abilities (such as the ability to walk independently), increased tolerance to feeding, and enhanced ability to perform activities of daily life. Additionally, relative stability in biochemical markers, such as sustained control of blood ammonia and methylmalonic acid levels compared to baseline, was also considered objective evidence of clinical benefit.

### Genetic mutation analysis

After informed consent was obtained from the guardians, 2 mL of blood was collected from each child and both parents into EDTA-anticoagulant tubes. Genomic DNA was extracted and stored at -20 °C for subsequent analysis. The entire mutational analysis was performed by Beijing MyGenostics Co., Ltd. Pathogenic variants detected via next-generation sequencing were verified by using conventional Sanger sequencing, and the corresponding variant sites were subsequently examined in the parental DNA.

The reference sequences for the MMACHC gene (NG_013378.1) and the MUT gene (NG_007100.1) were obtained from GenBank. All the identified variants were compared with these reference sequences and with entries in the Human Gene Mutation Database (HGMD) to determine pathogenicity. Variant pathogenicity was evaluated and classified according to the ACMG/AMP clinical practice guidelines. For variants already reported, their clinical significance was further confirmed by the ClinVar database (**Supplementary Tables 1**, **2**).

### Statistical analysis

Statistical analysis was performed using IBM SPSS 26.0 and GraphPad Prism 9.5.0. Categorical variables were expressed as n (%) and compared using the chi-square test or Fisher’s exact test, as appropriate. Continuous variables were expressed as Median (IQR) and compared between groups (isolated MMA vs combined MMA with hyperhomocysteinemia) using the Mann-Whitney U test, as data were not normally distributed according to the Shapiro-Wilk test. A two-sided P value < 0.05 was considered statistically significant. To account for multiple comparisons across the clinical, biochemical, and imaging parameters, the Benjamini-Hochberg procedure was applied to control the False Discovery Rate (FDR). Both unadjusted P-values and FDR-adjusted q-values were reported in the tables. A q-value < 0.05 was considered to indicate statistically significance after correction for multiple comparisons.

## Results

### Clinical manifestations

**Figure 1:**
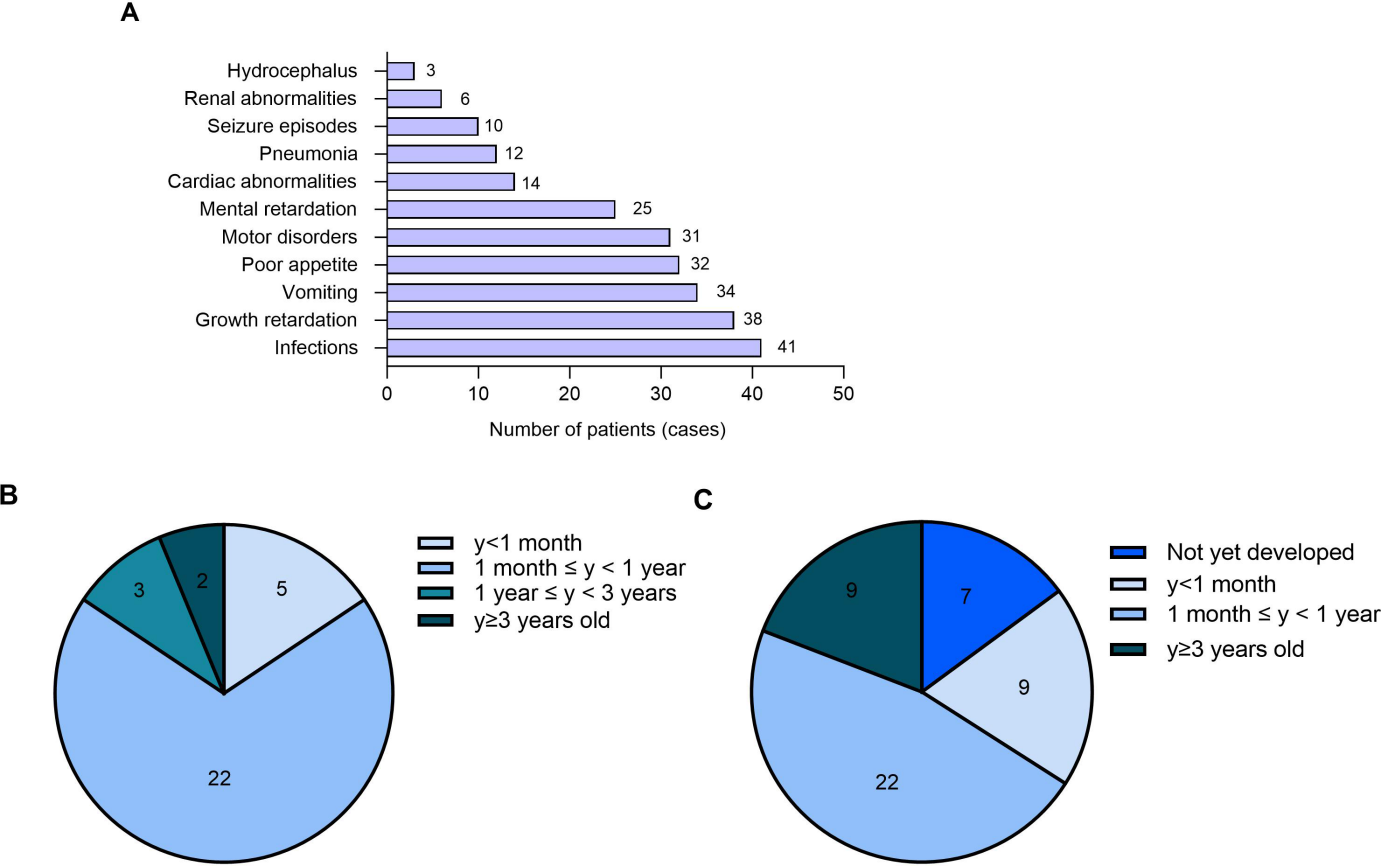
Clinical manifestations and age at onset in MMA patients. **(A)** Clinical manifestations at the onset of MMA diagnosis in children; **(B)** age at the onset of MMA in children with isolated MMA (n=32), defined as patients with methylmalonic aciduria and normal plasma homocysteine levels; **(C)** age at the onset of MMA in children with combined MMA with hyperhomocysteinemia (n=47), defined as patients with simultaneous elevations of methylmalonic acid and homocysteine in blood or urine.

As shown in **Figure 1A**, most of the children with MMA were referred to the outpatient clinic due to recurrent infections, failure to thrive, vomiting, feeding difficulties, motor impairment, and intellectual disability. Additionally, a small number of patients presented with cardiac dysfunction, seizures, renal anomalies, and hydrocephalus. A total of 51.9% of the patients (41/79) exhibited febrile prodrome with tachypnoea and cough.

As shown in **Table 1**, children with MMA commonly presented with infection, growth retardation, motor impairment, intellectual disability, feeding difficulties, and vomiting. Compared with the combined MMA with hyperhomocysteinemia group, vomiting was more frequently observed in children with isolated MMA. However, after applying the Benjamini-Hochberg procedure to account for multiple comparisons, only the prevalence of vomiting remained significantly different between the subtypes (q < 0.05). While a nominally higher frequency of cardiac abnormalities was observed in the combined MMA with hyperhomocysteinemia group, this did not reach statistical significance after FDR correction. For most other clinical manifestations, no significant differences were observed between the two groups.

**Table 1:** Clinical manifestations at onset of MMA diagnosis in children.

Clinical manifestations	Combined-type patients (47 cases)		Isolated-type patients (32 cases)		*χ* ^2^	*P*	*q*
	Number	Percentage (%)	Number	Percentage (%)			
Infection	23	48.9	18	56.3	0.408	0.523	0.575
Vomit	14	29.8	20	62.5	8.310	0.004	0.044
Poor appetite	15	31.9	17	53.1	3.554	0.059	0.216
Seizure	7	14.9	3	9.4	0.144	0.704	0.704
Movement disorder	15	31.9	16	50	2.612	0.106	0.292
Intellectual disability	12	25.5	13	40.6	2.005	0.157	0.345
Developmental delayed	20	42.6	18	56.3	1.431	0.232	0.425
Pneumonia	9	19.1	3	9.4	0.755	0.385	0.515
Hydrocephalus	3	6.4	0	0	0.735	0.391	0.515
Renal abnormality	5	10.6	1	3.1	0.648	0.421	0.515
Cardiac abnormality	12	25.5	2	6.3	4.854	0.028	0.154

P-values represented the unadjusted significance levels calculated by the Chi-square test or Fisher’s exact test for categorical variables. The q-values (FDR-adjusted) were calculated using the Benjamini-Hochberg procedure to account for multiple comparisons across the clinical, imaging, and genetic parameters. A q-value < 0.05 was considered statistically significant. For comparisons where the unadjusted P < 0.05 but q >0.05 (e.g., cardiac abnormalities), the results were interpreted as nominal trends rather than statistically significant differences after multiple-comparison adjustment.

### Age at onset

As illustrated in **Figures 1B, C**, most of the patients presented at the hospital between the 1^st^ and 12^th^ months of life. In the region in which newborn screening was performed, for isolated MMA patients, the age of onset ranged from 1 day to 3 years, with an average age of onset of 7.4 months being observed. Moreover, 13 patients (40.6%) were diagnosed through newborn screening, and 19 patients(59.4%)were identified via screening, compared with the assessment of clinical symptoms. For combined MMA with hyperhomocysteinemia, the age of onset ranged from 1 day to 17 years, with an average age of onset of 2.3 years being observed. In this group, 24 patients (51.1%) were diagnosed through newborn screening, and 23 patients (48.9%) were identified via screening, compared with the assessment of clinical symptoms. Among the 47 children with combined MMA with hyperhomocysteinemia, 7 patients (14.9%) remained asymptomatic and were identified only through newborn screening; all the others patients (85.1%) experienced symptoms. In contrast, all the children with isolated MMA developed clinical symptoms with a poor prognosis.

### Echocardiographic findings

**Figure 2:**
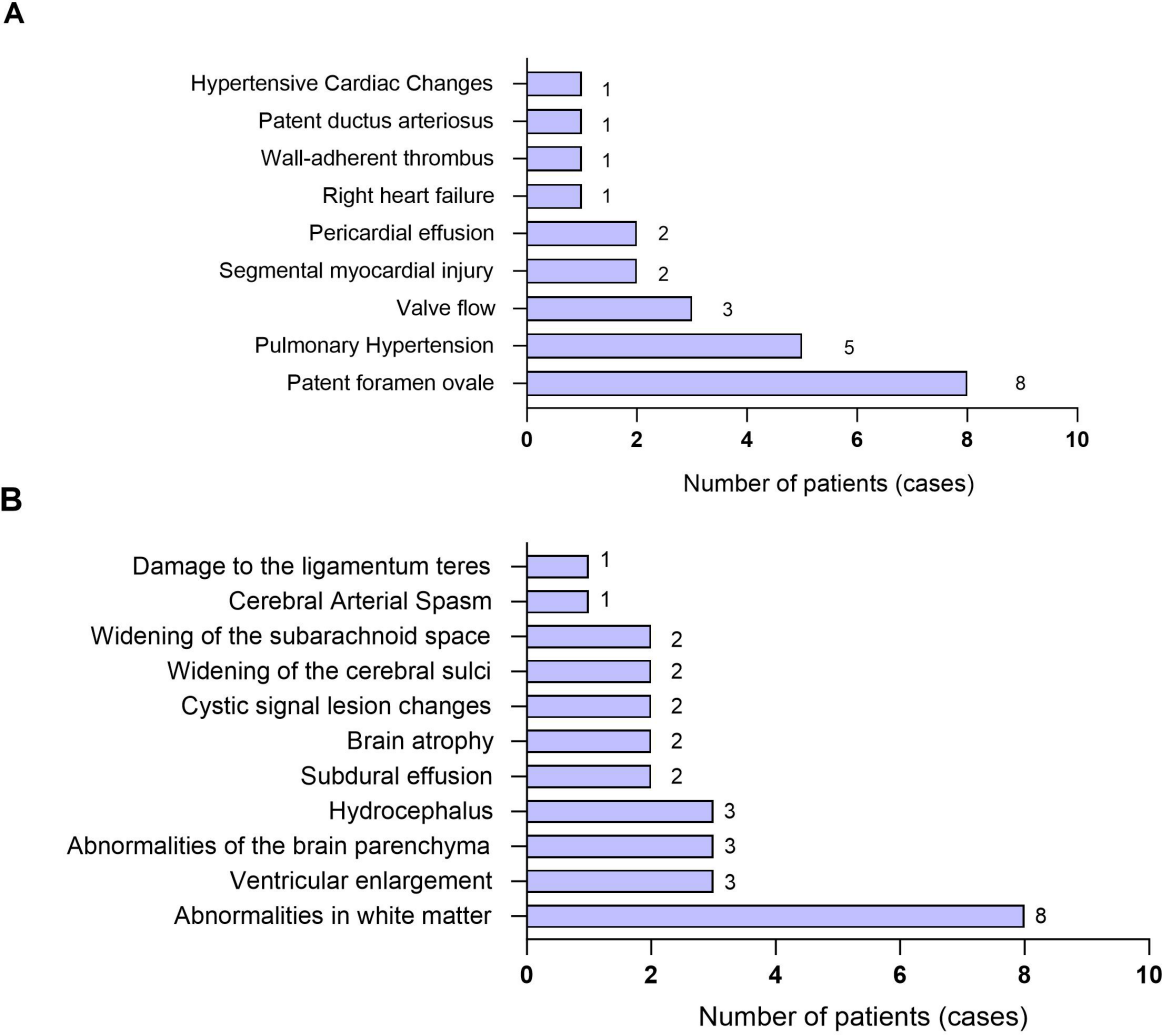
Cardiac echocardiography and cranial MRI findings in a child with MMA. **(A)** Cardiac structural abnormalities in a child with MMA; **(B)** cranial brain injury in a child with combined MMA with hyperhomocysteinemia.

Echocardiography (**Figure 2A**) revealed structural abnormalities in multiple cardiac regions. The most common disorders included patent foramen ovale (8 patients, 17.0%) and pulmonary hypertension (5 patients, 10.6%).

### Neuroimaging findings

Among the 47 children with combined MMA with hyperhomocysteinemia, 24 patients with neurologic manifestations underwent neuroimaging (**Figure 2B**). Abnormalities were detected in 17 patients. Typical findings included abnormal white matter signals, ventricular enlargement, myelination defects, and hydrocephalus.

Among the 32 children with isolated MMA, 16 patients underwent neuroimaging examination. Abnormalities were detected in 9 patients. All the patients exhibited multifocal cerebral lesions, abnormal pituitary height, and metabolic encephalopathic changes.

**Table 2** reported that among the 46 children with combined MMA with hyperhomocysteinemia, 28 patients had anemia (with a median value of 113.5 g/L). Among the 46 children in whom ALT levels were tested, 7 patients demonstrated abnormal levels (with a median value of 27.5 U/L). AST, serum creatinine, blood urea, and blood glucose levels were assessed in 45 patients, which revealed abnormal AST levels in 24 patients (with a median value of 42 U/L), an abnormal serum creatinine concentration in 3 patients (with a median value of 24.7 μmol/L), an abnormal blood urea concentration in 7 patients (with a median value of 3.4 mmol/L), and an abnormal blood glucose concentration in 1 patients (with a median value of 4.9 mmol/L). Plasma ammonia concentration was tested in 30 patients, with 1 case of hyperammonemia (with a median value of 40.5 μmol/L). Blood lactate levels were tested in 25 patients, with 21 cases of hyperlactatemia (with a median value of 3 mmol/L).

**Table 2:** Laboratory reports in children with MMA.

General biochemical tests	Combined-type patients			Isolated -type patients			*P* ^a^	*q* ^c^
	Number of individuals tested	Median (IQR)	Abnormalities, n (%)	Number of individuals tested	Median (IQR)	Abnormalities, n (%)		
Hb, g/L	46	113.5 (104.8, 120)	28 (60.9)	31	107 (94, 120)	21 (67.7)	0.194	0.323
ALT, U/L	46	27.5 (17.0, 36.3)	7 (15.2)	26	23 (17.8, 33.5)	5 (19.2)	0.639	0.639
AST, U/L	45	42 (33, 57)	24 (53.3)	26	38.5 (31.8, 49.8)	11(42.3)	0.595	0.639
Serum creatinine, μmol/L	45	24.7 (18.1, 33.8)	3(6.7)	28	23 (17.1, 30.2)	0	0.408	0.544
BUN, mmol/L	45	3.4 (2.0, 4.7)	7(15.6)	29	4.1 (3.1, 6.3)	4 (13.8)	0.032	0.256
Blood glucose, mmol/L	45	4.9 (4.4, 5.3)	1 (2.2)	32	5.1 (4.4, 6.9)	11 (34.4)	0.202	0.323
Blood lactate, mmol/L	25	3 (2.4, 4.3)	21 (84.0)	21	2.6 (2.2, 3.2)	18 (85.7)	0.171	0.323
Blood ammonia, μmol/L	30	40.5 (31, 57)	1 (3.3)	24	51 (44, 61)	1 (4.2)	0.094	0.323

ALT, alanine aminotransferase; AST, aspartate aminotransferase; BUN, blood Urea Nitrogen; Hb, hemoglobin; IQR, interquartile range.

a *P* values for continuous variables were calculated using the Mann-Whitney U test; for categorical variables, P values were calculated using the Chi-square test or Fisher’s exact test.

c *q* values represented the false discovery rate (FDR) adjusted P-values using the Benjamini–Hochberg procedure to account for multiple comparisons. A q value < 0.05 was considered statistically significant after multiple-comparison correction.

Among the children with isolated MMA, 21 patients had anemia (with a median value of 107 g/L), 5 patients exhibited abnormal ALT levels (with a median value of 23 U/L), 11 patients exhibited abnormal AST levels (with a median value of 38.5 U/L), 4 patients exhibited abnormal blood urea levels (with a median value of 4.1 mmol/L), 11 patients exhibited abnormal blood glucose levels (with a median value of 5.1 mmol/L), 1 patient demonstrated elevated plasma ammonia levels (with a median value of 51 μmol/L), and 18 patients exhibited elevated blood lactate levels (with a median value of 2.6 mmol/L). Hyperglycemia was significantly more common in children with isolated MMA, whereas no other biochemical indicators were significantly different between those with isolated MMA and those with combined MMA with hyperhomocysteinemia.

### Genetic mutation analysis in MMA patients

**Figure 3:**
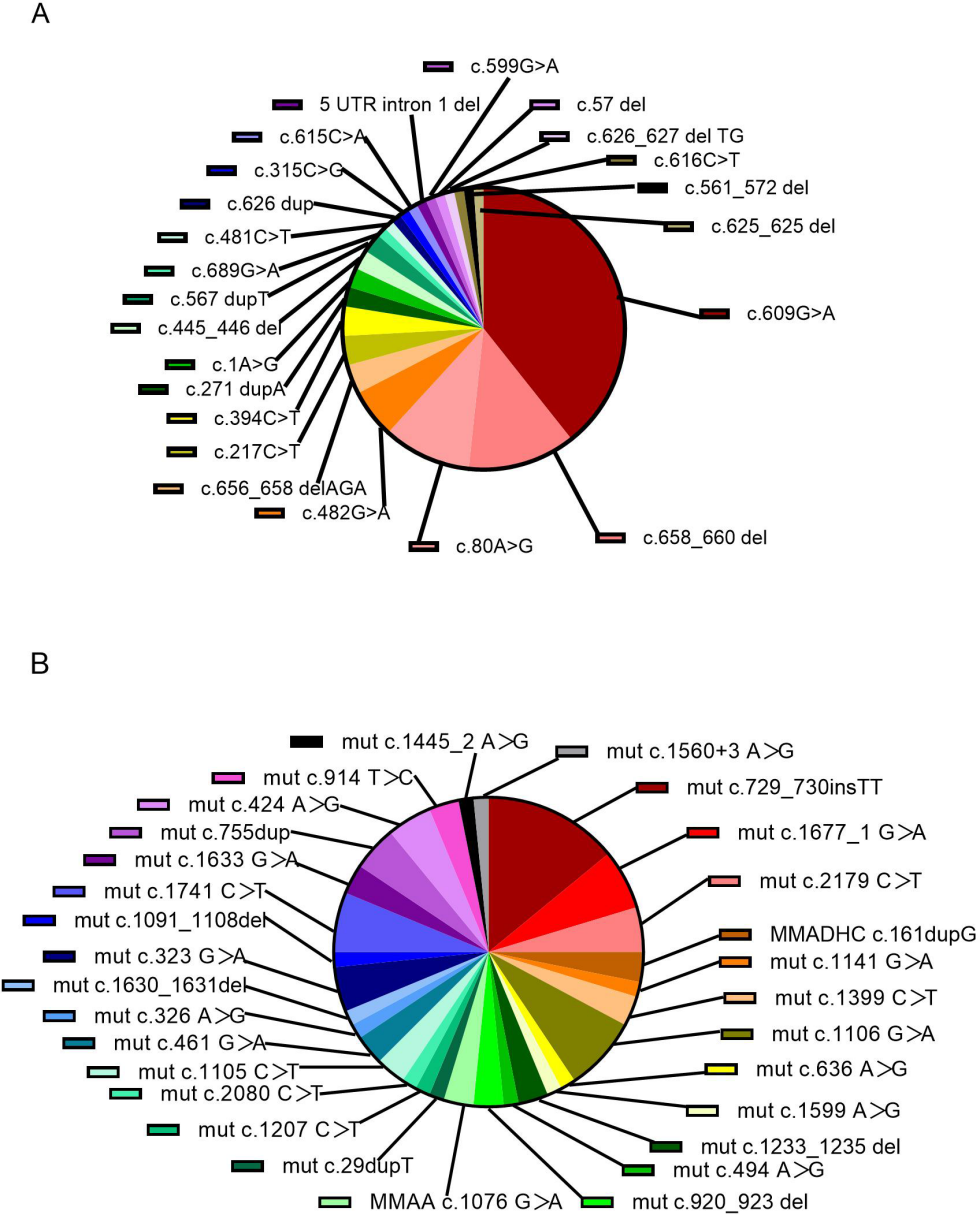
Types of MMA gene mutations. **(A)** Distribution of mutation types in patients with combined MMA with hyperhomocysteinemia (n=47). This group was defined as patients exhibiting simultaneous elevations of methylmalonic acid and homocysteine in blood or urine. **(B)** Distribution of mutation types in patients with isolated MMA (n=32). This group was defined as patients with elevated methylmalonic acid levels but normal homocysteine levels.

As shown in **Figure 3A**, 89 variant alleles were identified in the combined MMA with hyperhomocysteinemia cohort, corresponding to 23 distinct pathogenic variants. The mutational spectrum of combined MMA with hyperhomocysteinemia was dominated by *c.609G>A* and *c.658_660del*. The findings regarding isolated MMA are summarized in **Figure 3B**. A total of 64 mutant alleles were detected, encompassing 29 different variants. *Mut c.729_730insTT* was the most prevalent mutation detected in isolated MMA. Precise genotyping was performed on the data obtained from the 79 MMA patients (**Table 3**). Among the 47 patients with combined-type MMA, the *cblC* subtype (*MMACHC* gene mutation) was the predominant type, accounting for all 47 cases (100%). Among 32 patients with isolated MMA, *MMUT* gene mutations were the primary cause, comprising the *mut^0^*type (25 patients, 78.1%) and the *mut^-^*type (5 patients, 15.6%). Additionally, one case (3.1%) of the *cblD* type (*MMAA* gene mutation) and one case (3.1%) of the *cblA* type (*MMADHC* gene mutation) were identified. Among children with the combined phenotype, since the *MMACHC c.609G>A* mutation is the most common mutation in patients with the *cblC* phenotype, we compared clinical phenotypes and found that clinical manifestations of infection were the most common, followed by growth retardation, movement disorder, and poor appetite. Among children with the isolated MMA, since the *MMUT c.729_730insTT* mutation is the most common mutation in patients with the mut_0_ subtype, we observed that vomiting was the most common clinical manifestation, followed by feeding difficulties and infections.

**Table 3:** Distribution of MMA genotypes by clinical subtype.

Subtype	Disease-causing gene	Genetic subtype	Number of cases (n)	Percentage (%)
Combined-type patients (n=47)	MMACHC	cbIC	47	100.0
Isolated-type patients (n=32)	MUT	mut^0^	25	78.1
	MUT	mut^-^	5	15.6
	MMADHC	cbID	1	3.1
	MMAA	cbIA	1	3.1

### Hospitalization and prognosis

**Figure 4:**
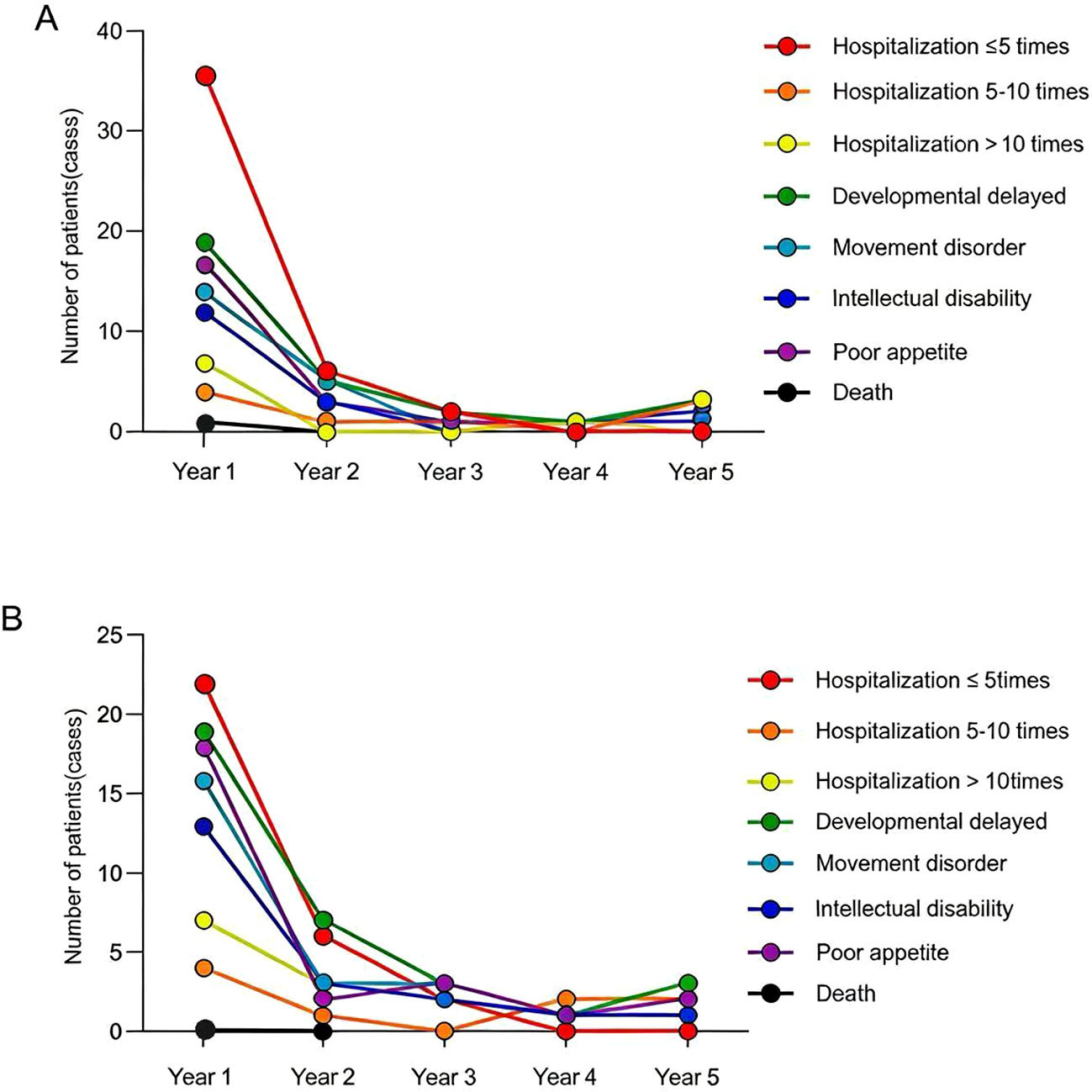
Analysis of hospitalization patterns in MMA patients for 5 years. **(A)** Five-year hospitalization patterns of patients with combined MMA with hyperhomocysteinemia (n=47); **(B)** five-year hospitalization patterns in patients with isolated MMA (n=32).

As shown in **Figures 4A, B**, compared with children with combined MMA with hyperhomocysteinemia, children with isolated MMA demonstrated more severe disease and required significantly more hospital admissions per year. After standard management, (including fluid resuscitation, acid-base correction, metabolic stabilization, and rehabilitative therapy), approximately half of the patients demonstrated improvements in motor and cognitive deficits. Nevertheless, many patients still retained variable degrees of motor and intellectual disability. One child with combined MMA with hyperhomocysteinemia died from severe hydrocephalus. Compared with patients who were diagnosed only after the onset of symptoms, those diagnosed through newborn screening demonstrated markedly better long-term outcomes, thus indicating the importance of early diagnosis.

## Discussion

MMA is a complex metabolic disorder characterized by significant clinical and genetic heterogeneity. Advances in high-throughput sequencing and treatment strategies have made early diagnosis possible; however, the long-term prognosis for many patients remains poor. Due to its diverse genotypes and variable phenotypes, MMA often affects multiple organs and is frequently misdiagnosed, resulting in irreversible brain damage before diagnosis. This leads to persistent motor and intellectual disabilities, severely impacting quality of life (21, 22). In addition, given the heavy financial burden on families, prenatal diagnosis (such as amniotic fluid enzyme activity testing or metabolite analysis) to prevent the birth of affected children is an effective measure to alleviate this burden (23, 24), even though these tests are not yet routinely performed in clinical practice. Liver transplantation and combined liver-kidney transplantation (CLKT) have been proposed as enzyme replacement therapies for MMA (25). Although liver transplantation improved the quality of life and metabolic stability of patients, it did not completely prevent the occurrence of brain damage, optic neuropathy, or cardiac disease (26). No patients in this cohort have yet undergone liver transplantation, and the clinical value of transplantation in this region remains to be further evaluated.

Newborn screening (NBS) is key to early detection. Of the 79 patients included in the study, 37 were diagnosed through newborn screening, while 42 were diagnosed after the onset of symptoms. Clinically, patients with symptom onset before 1 year of age are defined as early onset and patients with symptoms occurring after 1 year of age are defined as late onset, with early onset patients being associated with more severe manifestations and poorer prognosis (27, 28). To better contextualize these findings, current results were compared with recent studies in China. A multicenter study reported that MMA patients in China were typically characterized by early onset, high rates of metabolic decompensation, and poor prognosis, with significant subtype-related differences between cblC and mut forms (29). Similarly, data from a neonatal screening cohort demonstrated that MMA was associated with multisystem involvement, including neurodevelopmental delay and metabolic abnormalities (30). In addition, previous studies demonstrated that early-onset MMA accounted for the majority of cases and was often associated with more severe clinical outcomes (31). Consistent with these findings, current cohort also showed a predominance of early-onset disease and significant clinical heterogeneity. Between 2014 and 2023, newborn screening (NBS) for MMA in China underwent a gradual process of development. Beginning around 2014, tandem mass spectrometry (MS/MS) technology was gradually expanded from pilot programs in certain regions to multiple provinces, thereby improving detection rates and facilitating early diagnosis. In recent years, screening coverage and standardization were further improved, particularly in economically developed regions. However, several challenges remain: first, there are significant disparities in screening coverage and the distribution of medical resources across different regions; second, access to confirmatory genetic testing is limited in some areas, which may delay diagnosis; third, there is a lack of unified standards for follow-up management and long-term monitoring.

Despite standard metabolic interventions, neurodevelopmental challenges remain a major long-term issue for patients with MMA. The accumulation of MMA in the cerebral cortex causes oxidative damage and neuroinflammation, leading to impaired cognition and memory (32, 33). Typical MRI observations of MMA revealed a white matter swelling and signal abnormalities, as well as corpus callosum thinning, hydrocephalus, and signal changes in the basal ganglia (34). These observations were tightly associated with developmental delays (35). Recurrent infections, developmental delays, motor impairments, and intellectual disabilities were the primary characteristics of this group. Additional features included recurrent vomiting, jaundice, and cutaneous cyanosis (28, 36). Skin manifestations such as perioral dermatitis were relatively rare (37). Children with MMA might demonstrate a decrease in hemoglobin levels, as well as thrombocytopenia, hyperammonemia, and elevated lactate levels (38).

In recent years, although the survival rate of MMA patients was improved, their long-term prognosis remained unfavorable. Currently, standard management strategies for MMA primarily include limited protein intake, L-carnitine supplementation, the selective use of metronidazole and vitamin B12 therapy tailored to specific subtypes of MMA. Vitamin B12 supplementation may significantly improve the neurological prognosis in some patients, but there is considerable heterogeneity in response to the treatment among patients, and the underlying mechanisms remain unclear (2, 39, 40). Long-term nutritional interventions require the use of specially formulated infant formula to maintain total protein intake (41), with the proportion of natural protein adjusted according to individual needs (42). For transplant recipients, monitoring acid-base balance and infections during the perioperative period is critical (43). Survivors often face long-term complications such as anemia, renal insufficiency, and psychological and behavioral abnormalities (10, 44). During an acute metabolic crisis associated with MMA, impaired mitochondrial energy metabolism and gluconeogenesis may occur, leading to hypoglycemia (45, 46). Under the combined effects of hypoglycemia and metabolic stress, the patient body may activate counterregulatory hormonal responses, including the increase in glucagon, epinephrine, and growth hormone, to promote glycogenolysis and gluconeogenesis while to simultaneously induce insulin resistance, potentially resulting in stress-induced hyperglycemia (47, 48). In China, based on this center’s clinical experience, carglumic acid is commonly used to treat patients with isolated MMA, while nitrogen-removing agents are rarely used only in the treatment of hyperammonemia secondary to MMA.

In this study, 23 distinct gene mutations were identified in the combined MMA with hyperhomocysteinemia cohort, with the spectrum being dominated by *c.609G>A*, followed by *c.658_660del* and *c.80A>G*. The *c.609G>A* is a nonsense mutation involving the replacement of guanine with adenine at nucleotide 609, thereby resulting in the premature termination of the tryptophan codon and the subsequent loss of MMACHC protein function. Moreover, *c.658_660del* is an in-frame deletion that removes three nucleotides (AAG) at positions 658 to 660, thus resulting in the loss of a lysine residue at position 220 and leading to the partial loss of MMACHC protein stability and cobalamin-binding capacity. Furthermore, *c.80A>G* is a missense mutation involving the replacement of adenine with guanine at nucleotide 80, thus resulting in a substitution of glutamine by arginine at position 27 and likely impairing MMACHC protein folding and enzymatic function. In isolated MMA, 29 different mutations were detected, and the most frequently observed mutation was mut *c.729_730insTT*, which was observed nine times and represented a hotspot mutation among Chinese patients with MMUT deficiency. Additionally, the mut type showed a higher proportion of metabolic crises compared to other types, suggesting that the patients carrying this mutation were associated with earlier onset and more severe phenotypes. Common clinical manifestations included vomiting, feeding difficulties, and recurrent infections. These findings suggested that the *c.729_730insTT* mutation was potentially associated with more severe clinical manifestations in this cohort. Therefore, newborn screening may significantly improve early diagnosis rates, which is highly beneficial for enhanced monitoring and early intervention (2, 49, 50).

This study has several limitations. First, as a retrospective, single-center study, selection bias is inevitable, which limits the generalizability of the findings to the broader MMA population. The limited sample size (N = 79) further hinders robust statistical analysis of genotype-phenotype associations and prognostic predictors, meaning that these conclusions should be regarded as descriptive rather than definitive. Second, this study spans a decade (2014–2023), during which there were significant changes in the prevalence of newborn screening (NBS) and laboratory techniques in China; this temporal variation, combined with the fact that affected children were evaluated across multiple clinical centers, precludes consistent and continuous comparisons of treatment efficacy. Consequently, incomplete biochemical and nutritional data (e.g., plasma amino acid, acylcarnitine, or urinary organic acid profiles) limit our ability to make robust causal inferences regarding the impact of specific interventions on long-term prognosis. Third, the absence of standardized prognostic assessment metrics and a prospective healthy control group preclude rigorous quantification of disease-specific effects. In future prospective studies, the age and sex-matched healthy children as a control group should be included for more rigorously quantification of the disease impact. Additionally, this study did not systematically collect data on the patients’ nutritional status at diagnosis and post-treatment (e.g., body mass index and age and sex-adjusted height percentiles); consequently, we were unable to perform a stratified analysis of nutritional status, which was clearly a limitation of this study.

## Conclusion

In summary, this study provides a comprehensive description of the clinical, biochemical, and genetic characteristics of MMA in Shandong Province, China. Our findings highlight that MMA is a multisystemic disorder characterized by significant phenotypic and genotypic heterogeneity, frequently affecting the nervous, circulatory, and urinary systems. The observed clinical variability is closely associated with age at onset and the timeliness of diagnosis; newborn screening appears to be associated with a more stable metabolic profile and better clinical outcomes. Early identification through expanded newborn screening, followed by standardized metabolic management and long-term follow-up, remains the cornerstone of improving outcomes. These findings contribute to a deeper understanding of the regional disease burden and indicate that, while common mutations provide valuable clinical insights, further large-scale, multicenter prospective studies are needed to validate these associations and refine their predictive utility in clinical practice.

## Data Availability

The datasets presented in this study can be found in online repositories. The names of the repository/repositories and accession number(s) can be found in the article/**Supplementary Material**.
