# Supplementary material for: Clinical and genotypic analysis of 79 children with methylmalonic acidemia: a retrospective single-center study in China
Source: Front Endocrinol (Lausanne). 2026 Jun 15;17:1806231. doi: 10.3389/fendo.2026.1806231 (PMC13310777; doi:10.3389/fendo.2026.1806231)
Supplement: Supplementary file 1 [file Table1.docx]

**Supplementary Table S1:** Genetic variants in patients with Combined MMA with hyperhomocysteinemia

| **Gene** | **c.DNA** | **p.AA** | **Pathogenicity** |
| --- | --- | --- | --- |
| *MMACHC* | c.609G>A | p.W203X | Pathogenic |
| *MMACHC* | c.658_660 del | p.K220del | Pathogenic |
| *MMACHC* | c.80A>G | p.Q27R | Pathogenic |
| *MMACHC* | c.482G>A | p.R161Q | Pathogenic/Likely pathogenic |
| *MMACHC* | c.656_658 delAGA | p.219_220del | Pathogenic |
| *MMACHC* | c.217C>T | p.R73* | Pathogenic |
| *MMACHC* | c.394C>T | pR132X | Pathogenic |
| *MMACHC* | c.271 dupA | p.R91kfs*14 | Pathogenic |
| *MMACHC* | c.1A>G | p.M1V | Pathogenic |
| *MMACHC* | c.445_446 del | p.C149Hfs*32 | Pathogenic |
| *MMACHC* | c.567 dupT | p.I190Yfs*13 | Pathogenic |
| *MMACHC* | c.689G>A | p.R230Q | Uncertain Significance |
| *MMACHC* | c.481C>T | p.R161* | Pathogenic |
| *MMACHC* | c.626 dup | p.T210dfs*35 | Pathogenic |
| *MMACHC* | c.315C>G | p.Y105X | Pathogenic |
| *MMACHC* | c.615C>A | p.Y205X | Pathogenic |
| *MMACHC* | 5 UTR intron1 del | - | Pathogenic |
| *MMACHC* | c.599G>A | p.W200X | Pathogenic |
| *MMACHC* | c.57 del | p.F19Lfs*57 | - |
| *MMACHC* | c.626_627 del TG | p.V209Dfs*35 | Pathogenic/Likely pathogenic |
| *MMACHC* | c.616C>T | p.R206W | Pathogenic/Likely pathogenic |
| *MMACHC* | c.561_572 del | P.Asp188_Ala191del | Likely Pathogenic |
| *MMACHC* | c.625_625 del | P.V209Dfs*35 | - |

**Reference Sequences:** *MMACHC* (NM_015506.3). Gene and protein nomenclature follow the HGVS (Human Genome Variation Society) guidelines.

**Pathogenicity Classification:** Variants were classified according to the ACMG/AMP 2015 guidelines. **Pathogenic/Likely Pathogenic:** Variants with strong evidence of disease-causing potential. **Uncertain Significance**: Variants where the evidence for pathogenicity is currently insufficient or conflicting. **Benign/Likely Benign**: Variants that are not expected to cause disease, often due to high frequency in the general population or lack of functional impact.

**Symbols:** “-” indicates not applicable or information not available in public databases (e.g., p.AA change for large structural deletions or regulatory variants).
