# Supplementary material for: Clinical and genotypic analysis of 79 children with methylmalonic acidemia: a retrospective single-center study in China
Source: Front Endocrinol (Lausanne). 2026 Jun 15;17:1806231. doi: 10.3389/fendo.2026.1806231 (PMC13310777; doi:10.3389/fendo.2026.1806231)
Supplement: Supplementary file 2 [file Table2.docx]

**Supplementary Table S2：**Genetic variants in patients with Isolated MMA

| **Gene** | **c.DNA** | **p.AA** | **Pathogenicity** |
| --- | --- | --- | --- |
| *MMUT* | c.729_730insTT | p.D244FS*39 | Pathogenic |
| *MMUT* | c.1677_1 G>A | P？ | Pathogenic |
| *MMUT* | c.2179 C>T | p.Arg727Ter | Pathogenic |
| *MMADHC* | c.161dupG | p.T55Nfs*5 | Pathogenic |
| *MMUT* | c.1141 G>A | p.G381R | Pathogenic |
| *MMUT* | c.1399 C>T | p.Arg467Ter | Pathogenic |
| *MMUT* | c.1106 G>A | p.R369H | Pathogenic |
| *MMUT* | c.636 A>G | p.K212K | - |
| *MMUT* | c.1599 A>G | - | - |
| *MMUT* | c.1233_1235 del | p.Ile412del | Pathogenic/Likely pathogenic |
| *MMUT* | c.494 A>G | p.D165G | Pathogenic |
| *MMUT* | c.920_923 del | p.F307SfsX6 | Pathogenic |
| *MMAA* | c.1076 G>A | p.R3539Q | Pathogenic/Likely pathogenic |
| *MMUT* | c.29dupT | p.L10Ffs*39 | Pathogenic |
| *MMUT* | c.1207 C>T | P.R403* | Pathogenic |
| *MMUT* | c.2080 C>T | p.Arg694Trp | Pathogenic |
| *MMUT* | c.1105 C>T | p.Arg369Cys | Pathogenic |
| *MMUT* | c.461 G>A | p.Arg154His | Benign/Likely benign |
| *MMUT* | c.326 A>G | p.Q109R | Likely pathogenic |
| *MMUT* | c.1630_1631del | p.Gly544Ter | Pathogenic |
| *MMUT* | c.323 G>A | p.Arg108His | Pathogenic |
| *MMUT* | c.1091_1108del | p.Y364Sfs*4 | Uncertain significance |
| *MMUT* | c.1741 C>T | p.R581* | Pathogenic |
| *MMUT* | c.1633 G>A | p. Asp545Asn | Uncertain significance |
| *MMUT* | c.755dup | p.H252Qfs*6 | Pathogenic |
| *MMUT* | c.424 A>G | p.T142A | Pathogenic |
| *MMUT* | c.914 T>C | p.Leu305Ser | Pathogenic |
| *MMUT* | c.1445_2 A>G | - | Pathogenic/Likely pathogenic |
| *MMUT* | c.1560+3 A>G | P？ | Uncertain significance |

**Reference Sequences:** *MMUT* (NM_000255.4). Gene and protein nomenclature follow the HGVS (Human Genome Variation Society) guidelines.

**Pathogenicity Classification:** Variants were classified according to the ACMG/AMP 2015 guidelines. **Pathogenic/Likely Pathogenic:** Variants with strong evidence of disease-causing potential. **Uncertain Significance**: Variants where the evidence for pathogenicity is currently insufficient or conflicting. **Benign/Likely Benign**: Variants that are not expected to cause disease, often due to high frequency in the general population or lack of functional impact.

**Symbols:** “-” indicates not applicable or information not available in public databases (e.g., p.AA change for large structural deletions or regulatory variants).
